# A Treat-and-Extend Regimen of Intravitreal Brolucizumab for Exudative Age-Related Macular Degeneration Refractory to Aflibercept: A 12-Month Result

**DOI:** 10.3390/ph16040562

**Published:** 2023-04-07

**Authors:** Wataru Kikushima, Yoichi Sakurada, Yoshiko Fukuda, Mio Matsubara, Yumi Kotoda, Atsushi Sugiyama, Kenji Kashiwagi

**Affiliations:** Department of Ophthalmology, Faculty of Medicine, University of Yamanashi, Yamanashi 409-3898, Japan

**Keywords:** age-related macular degeneration, polypoidal choroidal vasculopathy, intravitreal brolucizumab injection, treat-and-extend, switching

## Abstract

We aimed to investigate whether a treat-and-extend regimen of intravitreal brolucizumab (6.0 mg/0.05 mL) is effective for eyes with exudative age-related macular degeneration (AMD) refractory to aflibercept for 12 months. Sixty eyes from 56 patients receiving brolucizumab for exudative AMD refractory to aflibercept were included. Patients received a mean of 30.1 aflibercept administrations for a mean 67.9-month follow-up. All patients exhibited exudation on optical coherence tomography (OCT) despite regular 4–8 weeks of aflibercept administration. Visit 1 was scheduled at the same interval from the last aflibercept injection to the baseline. The treatment interval was extended or shortened by 1–2 weeks depending on the presence or absence of exudation on OCT. After switching to brolucizumab, the follow-up interval significantly extended at 12 months (before switching: 7.6 ± 3.8 weeks vs. at 12 months: 12.1 ± 6.2 weeks, *p* = 1.3 × 10^−7^). Forty-three percent of the eyes achieved a dry macula at 12 months after switching. However, the best-corrected visual acuity did not improve at any visit. Morphologically, the central retinal thickness and subfoveal choroidal thickness significantly decreased from baseline at 12 months (*p* = 3.6 × 10^−3^ and 1.0 × 10^−3^, respectively). Switching to brolucizumab can be considered to extend the treatment interval in eyes with exudative AMD refractory to aflibercept.

## 1. Introduction

Age-related macular degeneration (AMD), one of the leading causes of blindness in developed countries, accounts for 8.7% of all cases of blindness worldwide and the projected number of people affected by this disease has been estimated to increase to 288 million in 2040 worldwide [1,2,3,4,5,6]. Advanced AMD is classified into two subtypes, exudative AMD originating from macular neovascularization (MNV), and geographic atrophy, also known by the recent term “complete retinal pigment epithelium (RPE) and outer retinal atrophy” through optical coherence tomography (OCT) [7,8]. AMD is a chronic inflammatory eye disease with a combination of genetic and environmental factors and its hallmark is known as drusen [9,10]. Conventional drusen size is associated with the progression and development of advanced AMD [11]. In addition to drusen size and RPE abnormalities, the drusen type is associated with the progression of the advanced AMD subtype, including the MNV types [12,13,14,15,16,17]. In Asians, geographic atrophy is a rare phenotype of advanced AMD, and MNV is exclusively seen in daily clinical practice. The growing number of patients with exudative AMD and medical expenses has become a social problem and burden in the developed countries [18,19,20,21].

Several cytokines were identified in the tissue of macular neovascularization, including pigment epithelium-derived factor and vascular endothelial growth factor (VEGF) [22]. Among these cytokines, VEGF is a key factor in the development and progression of neovascular AMD [22,23,24]. The advent of VEGF inhibitors has revolutionized the treatment of exudative AMD, and intravitreal injection of VEGF inhibitors is currently an established treatment for exudative AMD [20,25,26]. Pegaptanib, the first commercially available VEGF inhibitor, was designed to bind and block the extracellular VEGF, VEGF_165_ [27]. However, in the VISION study using 1186 participants with neovascular AMD, all three dosing (0.3 mg, 1.0 mg, 3.0 mg) arms failed to improve best-corrected visual acuity (BCVA) at 54 weeks despite intravitreal injection, every six weeks, of pegaptanib [28]. Because of the lack of efficacy, this drug was eliminated from the market. Ranibizumab, the second commercially available VEGF inhibitor, with molecular size of 48 kDa, was designed to block all isoforms of VEGF-A and was approved by the Food and Drug Administration (FDA) in 2006 [29]. In large-scale randomized clinical trials (RCTs), monthly dosing of ranibizumab (0.5 mg/0.05 mL or 0.3 mg/0.05 mL) improved BCVA 5.4–6.6 letters and 8.5–11.3 letters during a 24-month follow-up. irrespective of MNV subtype in MARINA and ANCHOR trials, respectively [25,26]. Ranibizumab is the first drug to demonstrate visual improvement in eyes with exudative AMD in RCTs. Aflibercept, the third commercially available VEGF inhibitor approved by FDA in 2011, was designed to bind all VEGF-A isoforms, VEGF-B, and placental growth factor with molecular size of 97–115 kDa. It can deliver twice as many molecules per injection as ranibizumab (0.5 mg/0.05 mL). Therefore, aflibercept (2.0 mg/0.05 mL) was expected to have greater efficacy and longer durability on the resolution of exudation and activity in eyes with exudative AMD in comparison with ranibizumab (0.5 mg/0.05 mL). The VEGF-Trap Eye Investigation of Efficacy and Safety of Wet Age-related Macular Degeneration (VIEW 1) and VIEW 2 demonstrated that 8 weekly administrations of aflibercept (2.0 mg/0.05 mL) following a loading phase with 3 monthly injections, resulted in a similar visual improvement when compared with a 4-weekly administration of ranibizumab (0.5 mg/0.05 mL) [30]. VIEW 2 revealed that dry macula was achieved in 71.9% of cases at 52 weeks after administration of (2.0 mg/0.05 mL) aflibercept at an 8-week interval [30]. However, as reported in this clinical trial, several eyes show residual exudation including subretinal fluid (SRF) and intraretinal fluid (IRF) on OCT despite regular injections in less than 8 weeks in the real-world setting. In the era of VEGF inhibitors therapy, it is challenging to maintain a dry macula with an extension of follow-up interval, and the advent of new drugs with longer durability is desirable.

Brolucizumab, the 4th commercially available anti-VEGF drug, was approved for use in the treatment of exudative AMD by the FDA in 2019 [31,32]. It was also approved for diabetic macular edema (DME), and doses of brolucizumab taken every 6 weeks (6.0 mg/0.05 mL), with five loading doses, showed significant visual and anatomical improvement in eyes with DME in 52 weeks in Phase 3 trials, KESTREL and KITE [33]. Among commercially available VEGF inhibitors, brolucizumab has the smallest molecular weight, with 26 kDa. It can achieve stability and solubility in a single intravitreal injection with 0.05 mL [34]. At a dose of 6 mg, an equivalent molecular dose of brolucizumab has approximately 10 times greater concentration than aflibercept and approximately 20 times greater concentration than ranibizumab [34]. The smaller molecular size of brolucizumab enables more effective penetration of the retina and choroid than other anti-VEGF molecules including bevacizumab, ranibizumab, and aflibercept. In some animal experiments, brolucizumab showed higher exposure in the retina and choroid than ranibizumab and shorter time to maximum concentration in the retina than ranibizumab or aflibercept [31]. In phase 3 clinical trial of HAWK/HARRIER, intravitreal administration of (6.0 mg/0.05 mL) brolucizumab demonstrated similar visual gains to and better morphological improvement than the intravitreal administration of (2.0 mg/0.05 mL) aflibercept [35,36]. Although there remain safety concerns including intraocular inflammation (IOI), retinal vasculitis, and retinal vascular occlusion after intravitreal administration of brolucizumab, there is mounting evidence regarding incidence, treatment, and prophylaxis of intraocular inflammation [37,38,39,40,41,42,43,44,45,46]. Though these data from animal experiments and clinical trials suggest the possible advantage of brolucizumab over other anti-VEGF agents, few data reveal the efficacy of switching to brolucizumab from other anti-VEGF agents for neovascular AMD with persistent exudation, including SRF and IRF, despite multiple intravitreal injections.

In the present study, we investigated one-year efficacy of switching to intravitreal administration of 6.0 mg brolucizumab for exudative age-related macular degeneration refractory to aflibercept using a treat-and-extend regimen.

## 2. Results

### 2.1. Study Cohort

Sixty eyes from 56 patients were included in this study. The mean age was 76.2 ± 7.4, and 51 patients were male (85.0%). Forty eyes (66.7%) were treated for polypoidal choroidal vasculopathy (PCV) and 20 eyes (33.3%) were treated for neovascular AMD. Table 1 presents the baseline characteristics of the patients. The mean follow-up period before switching was 67.9 ± 34.5 months and the mean number of total injections was 30.1 ± 17.8. Fourteen patients with PCV had a history of photodynamic therapy (PDT) (Table 1) and none of eyes with neovascular AMD had a history receiving PDT.

### 2.2. Treatment Interval

**Figure 1:**
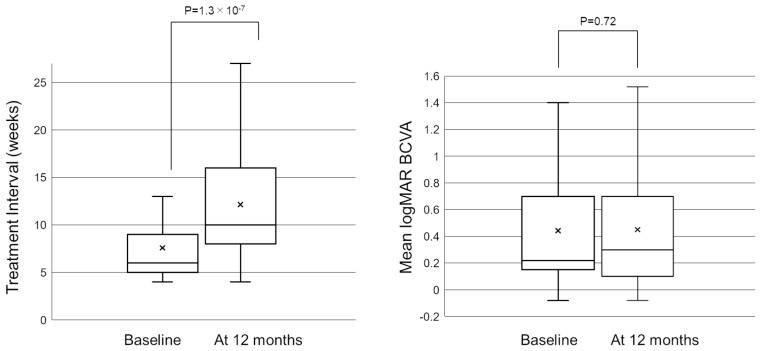
Box−and−whisker plot of mean and median treatment interval and best−corrected visual acuity (BCVA) before and after switching to brolucizumab. The “x” marks in the boxes indicate the mean treatment interval/BCVA. The horizontal lines in the boxes indicate the median treatment interval/BCVA, and the top/bottom edges of the boxes indicate the 25/75 percentile. The top/bottom edges of the whiskers indicate maximum/minimum values. (**Left**) At 12 months, the mean treatment interval significantly extended from 7.6 ± 3.8 weeks to 12.1 ± 6.2 weeks (*p* = 1.3 × 10^−7^). (**Right**) The mean logMAR BCVA was 0.44 ± 0.39 at baseline and was maintained to 0.45 ± 0.43 at 12 months after switching (*p* = 0.72). BCVA, best-corrected visual acuity; logMAR, logarithm of minimal angle resolution.

Figure 1 shows the mean treatment interval before and after switching to brolucizumab. After switching to brolucizumab, the mean treatment interval at 12 months was significantly longer than that before switching (7.6 ± 3.8 weeks at baseline, and 12.1 ± 6.2 weeks at 12 months, *p* = 1.3 × 10^−7^). Twenty-six eyes (43.3%) did not show any extension in the interval at 12 months. Twenty-seven (45.0%) eyes showed an extended interval of 4 weeks or more at 12 months. After switching, the patients received a mean of 6.15 ± 2.40 injections during 12 months, which was significantly fewer than those during 12 months before switching (mean of 6.87 ± 2.92 injections, *p* = 0.045).

### 2.3. Visual and Anatomical Outcomes

**Figure 2:**
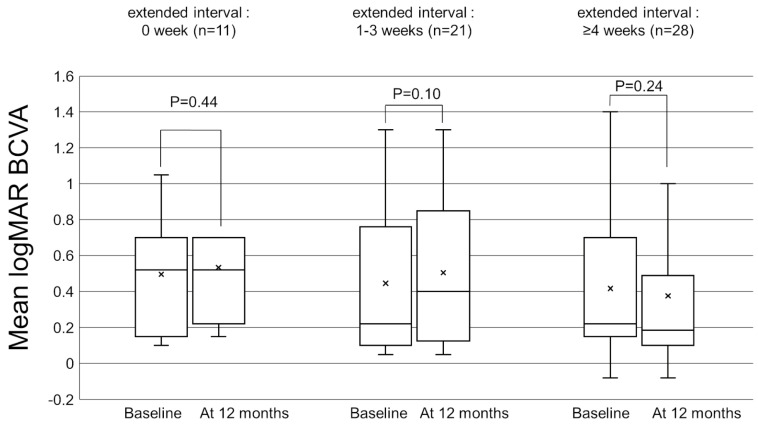
Box-and-whisker plot of mean and median best-corrected visual acuity (BCVA) before and after switching to brolucizumab according to the extended treatment intervals. The “x” marks in the boxes indicate the mean BCVA. The horizontal lines in the boxes indicate the median BCVA, and the top/bottom edges of the boxes indicate the 25/75 percentile. The top/bottom edges of the whiskers indicate maximum/minimum values. (**Left**) The mean change of BCVA in the patients with extended intervals of 0 weeks. (**Middle**) The mean change of BCVA in the patients with extended intervals of 1–3 weeks. (**Right**) The mean change of BCVA in the patients with extended interval of ≥4 weeks.

**Figure 3:**
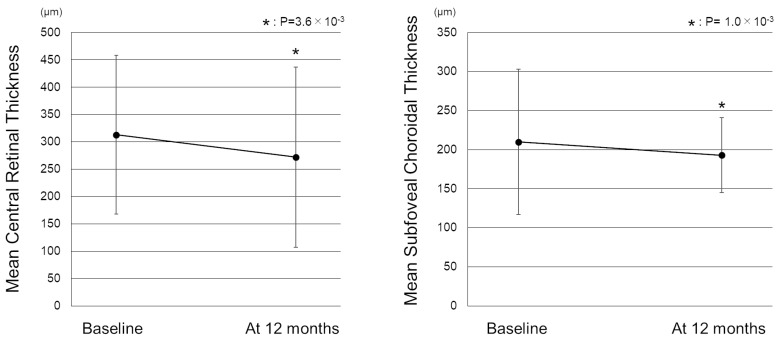
Change in the mean central retinal thickness and subfoveal choroidal thickness before and after switching to brolucizumab. (**Left**) The mean CRT significantly decreased from 313 ± 145 µm at baseline to 272 ± 165 µm 1 month after switching (*p* = 3.6 × 10^−3^). (**Right**) The mean SCT also significantly decreased from 210 ± 93 µm at baseline to 193 ± 48 µm at 12 months after switching (*p* = 1.0 × 10^−3^) CRT: central retinal thickness, SCT: subfoveal choroidal thickness.

**Figure 4:**
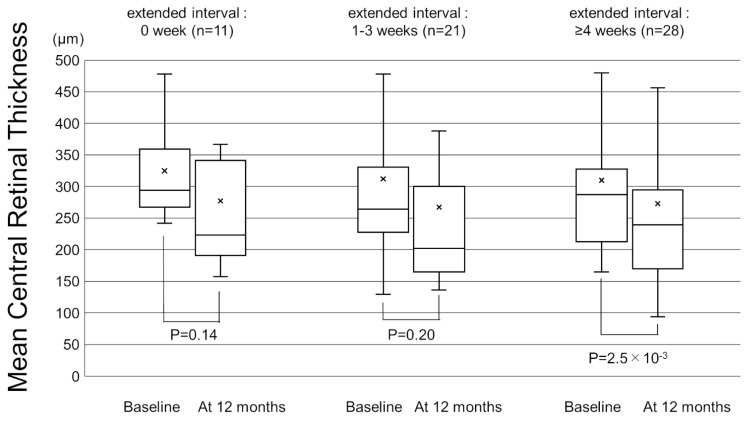
Box-and-whisker plot of mean and median central retinal thickness (CRT) on swept-source optical coherence tomography (SS-OCT) before and after switching to brolucizumab according to the extended treatment intervals. The “x” marks in the boxes indicate the mean CRT. The horizontal lines in the boxes indicate the median CRT, and the top/bottom edges of the boxes indicate the 25/75 percentile. The top/bottom edges of the whiskers indicate maximum/minimum values. (**Left**) The mean change of CRT in the patients with extended intervals of 0 weeks. (**Middle**) The mean change of CRT in the patients with extended intervals of 1–3 weeks. (**Right**) The mean CRT significantly decreased from baseline to 12 months in the patients with extended interval of ≥4 weeks.

**Figure 5:**
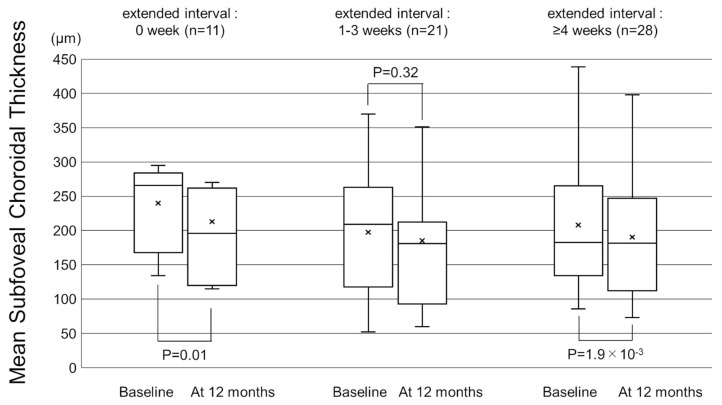
Box-and-whisker plot of mean and median subfoveal choroidal thickness (SCT) on swept-source optical coherence tomography (SS-OCT) before and after switching to brolucizumab according to the extended treatment intervals. The “x” marks in the boxes indicate the mean SCT. The horizontal lines in the boxes indicate the median SCT, and the top/bottom edges of the boxes indicate the 25/75 percentile. The top/bottom edges of the whiskers indicate maximum/minimum values. (**Left**) The mean SCT significantly decreased from baseline to 12 months in the patients with extended intervals of 0 weeks. (**Middle**) The mean change of SCT in the patients with extended intervals of 1–3 weeks. (**Right**) The mean SCT significantly decreased from baseline to 12 months in the patients with extended interval of ≥4 weeks.

Figure 1 shows the change in the mean best-corrected visual acuity (BCVA) before and after switching. Mean logarithm of the minimal angle resolution (logMAR) BCVA was maintained from 0.44 ± 0.39 at baseline to 0.45 ± 0.43 at 12 months after switching (*p* = 0.72). To investigate the correlation between visual outcomes and changes in the treatment intervals, we divided the patients into 3 groups according to the length of the extended treatment intervals (0 weeks, 1–3 weeks, and 4 weeks or more). The results showed no significant difference in mean BCVA from baseline to 12 months in each group (Figure 2). The mean central retinal thickness (CRT) on swept-source optical coherence tomography (SS-OCT) significantly decreased from 313 ± 145 µm at baseline to 272 ± 165 µm at 12 months after switching (*p* = 3.6 × 10^−3^, paired *t*-test, Figure 3). The mean subfoveal choroidal thickness (SCT) on SS-OCT also significantly decreased from 210 ± 93 µm at baseline to 193 ± 48 µm at visit 3 after switching (*p* = 1.0 × 10^−3^, paired *t*-test, Figure 3). Figure 4 and Figure 5 show the mean change of CRT/SCT on SS-OCT according to the length of the extended treatment intervals. Mean CRT significantly decreased from baseline to 12 months in the patients with extended intervals of ≥4 weeks (Figure 4). Mean SCT significantly decreased in the patients with extended intervals of 0 weeks and ≥4 weeks (Figure 5).

**Figure 6:**
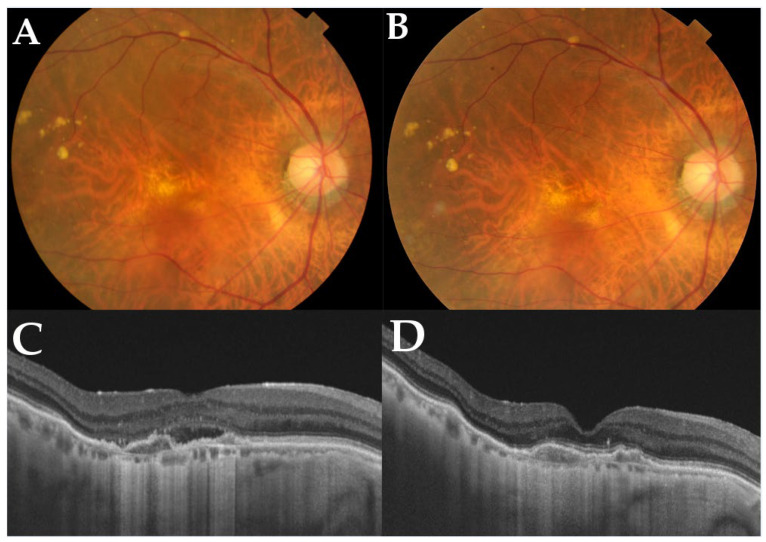
A representative case of an 82-year-old male with polypoidal choroidal vasculopathy in the right eye who switched to brolucizumab. Before switching, he had received a total of 50 injections during the follow-up period of 81 months. (**A**) At baseline, a color fundus photograph shows depigmentation at the juxtafoveal area. (**B**) At 12 months, a color fundus photograph shows no apparent changes compared with the baseline. (**C**) A horizontal OCT scan of the macula shows subretinal fluid and double-layer signs at baseline. After switching to brolucizumab, horizontal OCT scans of the macula at 12 months (**D**) show complete resolution of subretinal fluid accumulation. Best-corrected visual acuity in his right eye was improved from 0.15 logarithm of minimum angle resolution (logMAR) from the baseline to 0.10 loMAR at 12 months. The interval between the last aflibercept injection and the baseline was 5 weeks, which was extended to 8 weeks at 12 months.

Table 2 shows the number of patients showing exudation on SS-OCT before and after switching. At 12 months, 26 out of 60 eyes (43.3%) achieved a dry macula. Among the remaining 34 eyes with exudation, 25 eyes showed SRF, 6 eyes showed IRF, and 3 eyes showed both SRF and IRF. Figure 6 shows a representative case of switching to brolucizumab.

### 2.4. Adverse Events

During the study period, adverse events were seen in 12 eyes (20%). Among 12 eyes, 2 eyes showed mild anterior chamber cells and keratic precipitates, and 7 eyes showed mild posterior IOI including vitreous opacity and anterior vitreous cells. Topical steroids were administered to these patients with mild anterior or posterior IOI, and the IOIs were immediately resolved. Intravitreal brolucizumab injection was discontinued, and the patient was switched to aflibercept again from the next visit. Floaters were seen in 3 eyes (5%) of 3 patients, and these patients underwent funduscopic examination with pupil dilation; however, fundus examination showed no sign of IOI, and no additional treatment was undertaken. The symptoms resolved spontaneously in these patients.

## 3. Discussion

In the present study, we investigated whether intravitreal brolucizumab (6.0 mg) was effective for exudative AMD refractory to aflibercept using a treat-and-extend regimen for 12 months. Before the initiation of brolucizumab therapy, the follow-up interval of all patients was equal to or less than 8 weeks. The patients had a mean of 30 aflibercept injections at regular 4–8-week (mean 7.6 weeks) intervals before switching to brolucizumab. At 12 months from baseline, a dry macula was achieved in almost half of the eyes that switched to brolucizumab. Visit 1 was scheduled at the same interval between the last aflibercept injection and the baseline; a dry macula was achieved in 26.7% of eyes at visit 1, suggesting that one session of intravitreal injection of brolucizumab was more effective than aflibercept from a morphological point of view.

Although study design, treatment regimen, and study duration were different, several studies have investigated the efficacy of brolucizumab for persistent neovascular AMD [46,47,48,49,50,51,52,53,54]. Recently, Ueda-Consolvo et al. reported an 18-month follow-up study of switching to brolucizumab from aflibercept in 42 eyes with exudative AMD [50]. They reported that treatment intervals were significantly extended from 7.4 ± 1.4 weeks to 11.6 ± 2.6 weeks for type 1 macular neovascularization and from 6.9 ± 1.3 weeks to 11.7 ± 3.1 weeks for polypoidal choroidal vasculopathy. They also reported that IOI occurred in 16.7% (7/43) of study eyes. Similarly, the results in this study showed the significant extension of treatment intervals from 7.6 ± 3.8 weeks to 12.1 ± 6.2 weeks at 12 months, and IOIs occurred in 15.0% (9/60) of study eyes. These results also support the efficacy of brolucizumab for exudative AMD refractory to aflibercept and IOIs appeared in almost one out of six eyes among Japanese patients. The incidence of IOIs in these studies was relatively higher than that in a large cohort study conducted in the United States [55]. Although the exact reason was unknown, the differences in race, including pigmentation of RPE and the size of the study population, might affect the results.

Brolucizumab is a novel VEGF inhibitor with a molecular mass of 26 kDa, less than commercially available ranibizumab (48 kDa) and aflibercept (97–115 kDa). Owing to its high solubility, brolucizumab can be concentrated up to 120 mg/mL [29]. Therefore, the binding affinity of brolucizumab to VEGF is greater than that of aflibercept. Thus, brolucizumab has a therapeutic advantage because of its prolonged effect. This study’s treatment interval significantly extended from the first to the last interval at 12 months (from 6.15 ± 2.40 to 6.87 ± 2.92, *p* = 0.045). Several studies have reported the efficacy of brolucizumab for refractory exudative AMD in short-term results in the real-world [41,49,56,57,58,59,60,61,62]. In the present study, 45.7% (26 eyes) could not extend the treatment interval during 12-month follow-up. This means that almost half of poor responders to aflibercept therapy did not respond to a treat-and-extend regimen of brolucizumab. Although we can select various VEGF inhibitors with the advent of brolucizumab, we must recognize that treatment resistance concerns remain as well as safety concerns. and we must work on how to treat these patients.

In the present study, BCVA was almost similar between baseline and at 12 months, although half of the eyes achieved a dry macula. In a previous study, Enríquez et al. investigated the efficacy of switching to brolucizumab for neovascular AMD refractory to the prior anti-VEGF agents because of persistent fluid. They reported that there was no difference in mean VA of the patients with neovascular AMD prior to starting brolucizumab compared with after brolucizumab injections or at the final study evaluation [53]. The FLUID study revealed that the presence of SRF less than 200 µm at the fovea was tolerable concerning BCVA improvement in patients with neovascular AMD treated with ranibizumab [63]. However, the previous study period was relatively short (24 months). Therefore, assuming a long-standing visual prognosis in eyes with exudative AMD, complete resolution of SRF is preferable when treated with anti-VEGF agents. Further studies are needed to confirm these questions.

About SCT after treatment for exudative age-related macular degeneration, three monthly ranibizumab administrations induced a decrease in SCT by 6%, three monthly aflibercept administrations induced a decrease in SCT by 12–16% and photodynamic therapy (PDT) induced a decrease SCT by 20% [64,65,66,67,68]. A recent study reported that subfoveal choroidal thickness significantly decrease by 20% after 3 monthly brolucizumab injections, [69] suggesting 3 monthly administration of brolucizumab has a similar effect on choroidal thickness to PDT. The effect of brolucizumab on choroidal thickness might be associated with greater strength of exudation resolution.

This study had several limitations. Major limitations are the small number of patients and the retrospective nature of the study. To validate the present conclusions, a longer-duration study and/or a large-scale prospective study are needed. In addition, we only focused on the anatomical/functional outcomes after the administration of brolucizumab and did not evaluate specific molecules or immunological parameters. Therefore, we could not discuss the preceding pathologies at the molecular level.

In summary, treat-and-extend regimen using brolucizumab is effective for improving the anatomical outcomes in eyes with exudative AMD refractory to aflibercept.

## 4. Materials and Methods

### 4.1. Participants

A retrospective medical chart review was performed on consecutive patients with exudative AMD treated with 6.0 mg brolucizumab, initiating a treat-and-extend regimen from 1 September 2020 to 1 June 2021 in the Macula Clinic, Department of Ophthalmology, University of Yamanashi Hospital. This retrospective study was approved by the Institutional Review Board of the University of Yamanashi (approval number: 2485 approval date: 13 July 2021) and was conducted in accordance with the tenets of the Declaration of Helsinki. Written informed consent for treatment was obtained from all patients.

The inclusion criteria were as follows: (1) eyes with neovascular AMD or PCV, (2) eyes showing exudation including subretinal or intraretinal fluid despite regular intravitreal aflibercept (2.0 mg/0.05 mL) injections equal to or less than 8-week intervals, (3) eyes that completed the follow-up of at least 12 months from switching.

The exclusion criteria were as follows: (1) eyes with retinal angiomatous proliferation, myopic choroidal neovascularization, or choroidal neovascularization secondary to angioid streaks; (2) treatment-naïve eyes, and (3) eyes treated with brolucizumab using other treatment regimens including regular injections or as-needed injections; (4) eyes within a 12-month follow-up period; (5) eyes receiving other treatment including cataract surgery, sub-tenon triamcinolone acetonide injection during the study period.

### 4.2. Diagnosis

The diagnosis of neovascular AMD or PCV was made on multimodal imaging by comprehensive examination including color fundus photography using TRC-50DX (Topcon, Tokyo, Japan), SS-OCT (DR-1/Atlantis, Topcon, Tokyo, Japan), and fluorescein/indocyanine green angiography (FA/ICGA) using a confocal laser scanning system (HRA-2; Heidelberg Engineering, Dossenheim, Germany) as previously described [70]. Briefly, PCV showed aneurysmal dilation with or without branching vascular network on ICGA, and retinal pigment epithelium protrusion was observed corresponding to the aneurysmal orange red lesion on SS-OCT. The diagnosis of neovascular AMD was also made using angiography and SS-OCT. Neovascular AMD showed classic or occult choroidal neovascularization on FA without aneurysmal dilation on ICGA. Type 1 or 2 neovascularization was observed on SS-OCT. In eyes with Type 1MNV, subretinal hyperreflective materials were seen beneath the RPE on OCT, and in eyes with Type 2 MNV, hyperreflective materials were seen above the RPE on OCT. An OCT scan pattern consisted of both vertical and horizontal scans with a 9-mm length centering at the fovea.

### 4.3. A Treat-and-Extend Regimen

**Figure 7:**
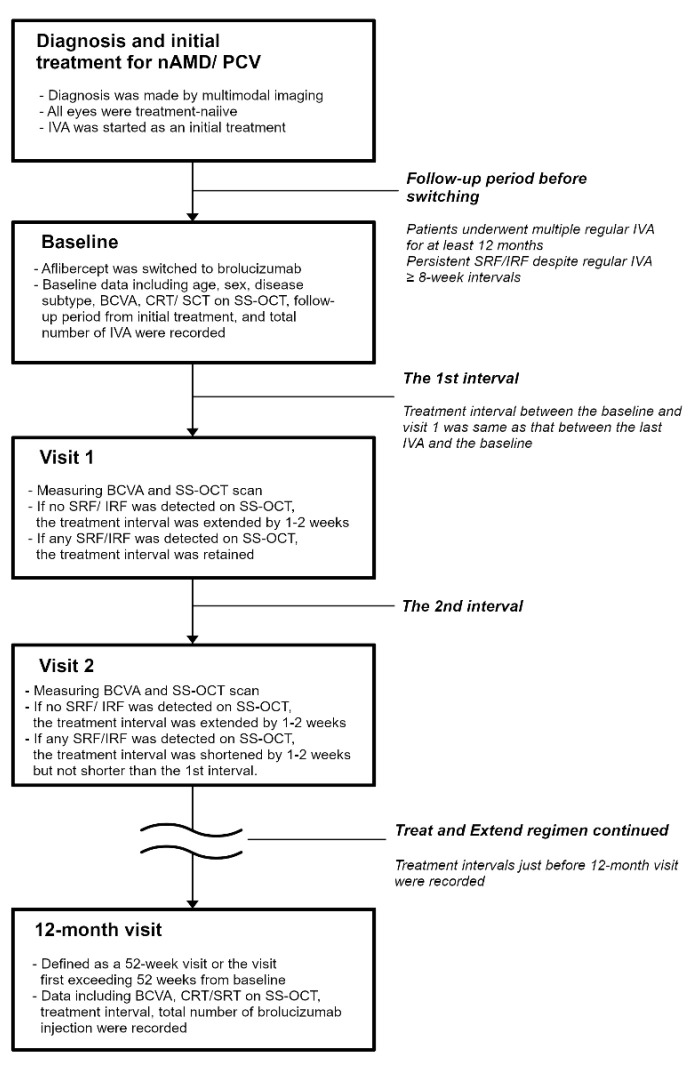
The flow-chart of treat-and-extendtreat-and-extend regimen. AMD, age-related macular degeneration; BCVA, best-corrected visual acuity; CRT, central retinal thickness; IRF, intraretinal fluid; IVA, intravitreal injection of aflibercept; SCT, subfoveal choroidal thickness; SRF, subretinal fluid; SS-OCT, swept-source optical coherence tomography.

The detailed flow-chart of the treat-and-extend regimen is shown in Figure 7. All patients had a treatment history of multiple regular aflibercept administrations for at least 12 months. At baseline, an intravitreal injection of brolucizumab (6.0 mg/0.05 mL) was administered in all eyes. The first visit (visit 1) was scheduled at the same interval between the last aflibercept injection and the baseline. The second visit (visit 2) was extended by 1–2 weeks based on one (Y.S) of the doctors’ discretion if there was no exudation including subretinal fluid and intraretinal fluid on SS-OCT during the first visit. If exudation was observed on SS-OCT, the interval between visits 1 and 2 was retained. Similarly, the next visits were extended by 1–2 weeks if there were no exudations including subretinal and intraretinal fluid on SS-OCT during the previous visits. If exudation was observed on SS-OCT, the next interval was shortened by 1–2 weeks, as the interval was extended at the previous visit. None of the intervals were designed to be shortened to less than the first interval. A “12-month visit” is defined as a 52-week visit or the visit first exceeding 52 weeks. All of the patients were instructed to make an immediate call in case of any abnormal symptoms after injection. Patients who reported symptoms of floater or blurred vision after the intravitreal injection were immediately seen in our hospital. 

### 4.4. Follow-Up Examination

At every visit, all patients underwent BCVA measurement using a Landolt chart, intraocular pressure measurement, slit-lamp biomicroscopy with or without 78D lens, color fundus photography, and SS-OCT using DR-1/Atlantis. Scan signal strength equal to or more than 6 was applied to all.

### 4.5. Statistical Analysis

Statistical analyses were performed using the Statflex 7 software (Artec Co., Ltd., Osaka, Japan). BCVA measured on a Landolt chart was converted into logMAR for statistical analyses. The paired *t*-test was used to determine the significance of the difference between the values before and after treatment. Statistical significance was set at *p*-value less than 0.05.

## Figures and Tables

**Table 1:** Baseline characteristics of the patients with exudative age-related macular degeneration treated with anti-vascular endothelial growth factor agents and switched to brolucizumab.

Number of Eyes	60
Age	76.2 ± 7.4
Male gender	51 (85.0%)
Disease subtype (nAMD:PCV)	20:40
Baseline BCVA	0.44 ± 0.39
Baseline CRT on SS-OCT (µm)	313 ± 145
Baseline SCT on SS-OCT (µm)	210 ± 93
Mean follow-up period (month)	67.9 ± 34.5
Mean number of total injections	30.1 ± 17.8
Number of the patients with the past history of PDT (%)	14 (23.3%)

AMD: age-related macular degeneration, BCVA: best-corrected visual acuity, CRT: central retinal thickness, nAMD: neovascular age-related macular degeneration, PCV: polypoidal choroidal vasculopathy, PDT: photodynamic therapy, SCT: subfoveal choroidal thickness, SS-OCT: swept source-optical coherence tomography, VEGF: vascular endothelial growth factor.

**Table 2:** Number of the patients with or without exudative change before and after switching to intravitreal injection of brolucizumab.

	Dry Macula	SRF Only	IRF Only	SRF and IRF
At baseline	0/60	51 (85.0%)	4 (6.7%)	5 (8.3%)
At Visit 1	16/60 (26.7%)	35 (58.3%)	6 (10.0%)	3 (5.0%)
At 12 months	26/60 (43.3%)	25 (41.7%)	6 (10.0%)	3 (5.0%)

IRF: intraretinal fluid, SRF: subretinal fluid.

## Data Availability

The data that support the findings of this study are not publicly available due to their containing information that could compromise the privacy of research participants but are available from the corresponding author upon reasonable request.
